# Intractable hiccup due to giant hydronephrosis: A rare case report and literature review

**DOI:** 10.1016/j.ijscr.2019.12.013

**Published:** 2019-12-13

**Authors:** Xiao-Xing Liao, Jiang-Hua Yang, Nian-Zeng Xing

**Affiliations:** a Department of Urology, Beijing Aerospace General Hospital, 100076 Beijing, China; b Department of Urology, The Chinese Academy of Sciences Cancer Hospital, Beijing, China

**Keywords:** Giant hydronephrosis, Intractable hiccups, Symptoms, Treatment, Case report

## Abstract

**Introduction:**

Giant hydronephrosis (GH) is a rare disease that is found in adult patients. Although there are some common symptoms associated with hydronephrosis, such as surrounding organ compressed, its rarer symptoms can render diagnosis very difficult, and treatment should also vary according to the cause.

**Presentation of case:**

We here report an 82-year-old man who was admitted to the hospital for repeated intractable hiccups. After B-ultrasound and CT examination, the patient underwent laparoscopy surgery, which was converted to open nephrectomy, and the patient’s intractable hiccup symptoms disappeared.

**Discussion:**

GH is a rare disease, and its symptoms are diverse. The more unusual symptoms of cystic hypertonic compression of surrounding organs, such as intractable hiccups, should be taken into account. GH is mainly diagnosed via ultrasound examination and CT scan. The choice of treatment for GH needs to be based on the etiology and renal function of hydronephrosis, and consider malignant lesions.

**Conclusion:**

Giant hydronephrosis can present rare symptoms as “intractable hiccups”. The selection of treatment should be made depending on the cause.

## Highlights

• A rare disease was reported in our case: giant hydronephrosis in adult patients.
• we reported for the first time that intractable hiccups due to hydronephrosis.
• Intractable Hiccup of the patient disappeared after nephrectomy.

## 1 Introduction

Hydronephrosis is a common clinical condition that is often caused by obstruction of the ureteropelvic junction, but giant hydronephrosis (GH) is rare, especially in adults. In adults, GH is defined by Sterling firstly in 1939 as the presence of more than 1 L of fluid in the renal pelvis, or kidney occupying the hemiabdomen across the midline [1].

GH may present with vague symptoms, including increased abdominal girth, nausea, fatigue, indigestion, and loss of weight, but, to the best of our knowledge, there have been no reports of intractable hiccups due to GH. We here present a rare case of an 82-year-old male patient who suffered from intractable hiccups due to GH, along with a review of the past decade. This case has been reported in accordance with the surgical case report guidelines (SCARE) criteria [2].

## 2 Case report

**Fig. 1:**
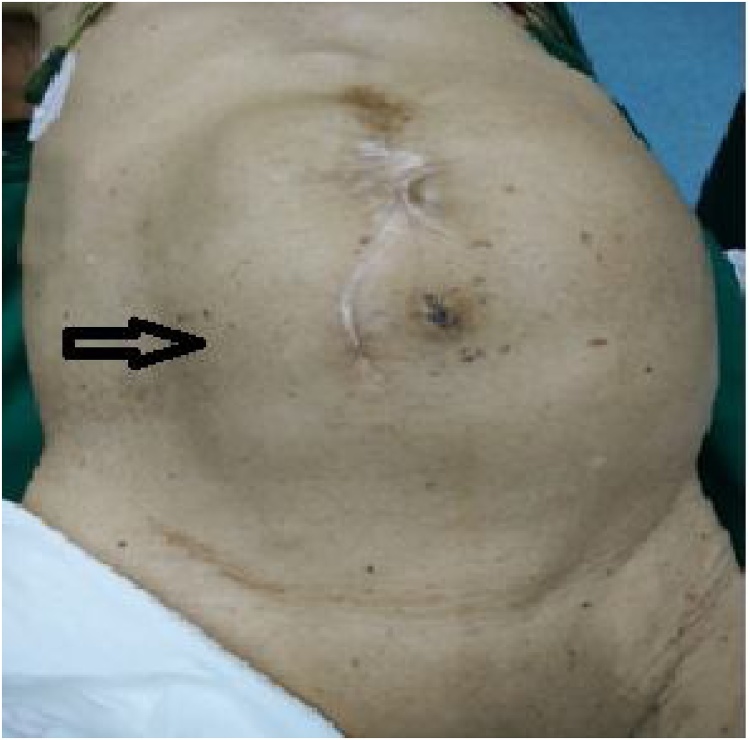
Preoperative picture of the patient showing the grossly distended abdomen caused by GH (black arrow).

**Fig. 2:**
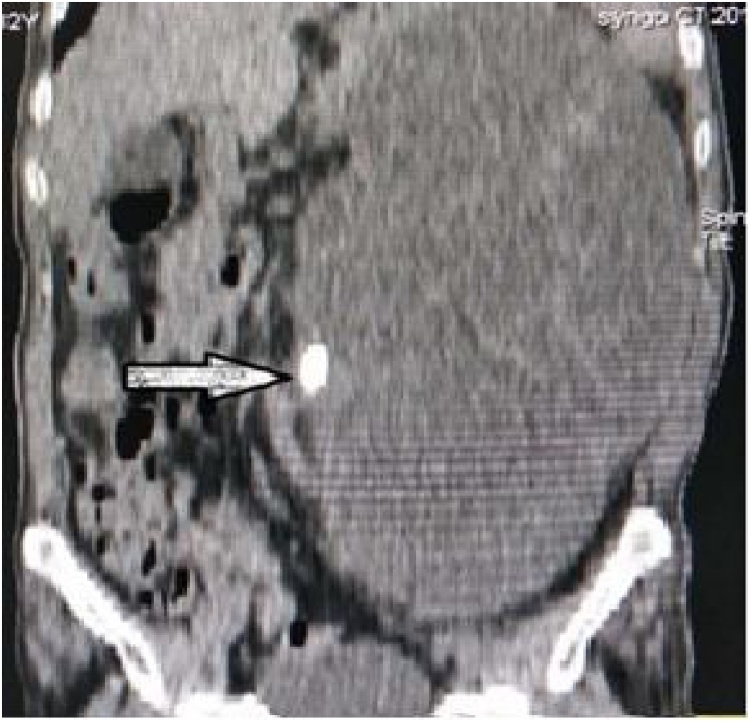
CT scan of the abdomen (coronal section) showing GH, and a high-density shadow (white arrow) located in the renal hilum region.

**Fig. 3:**
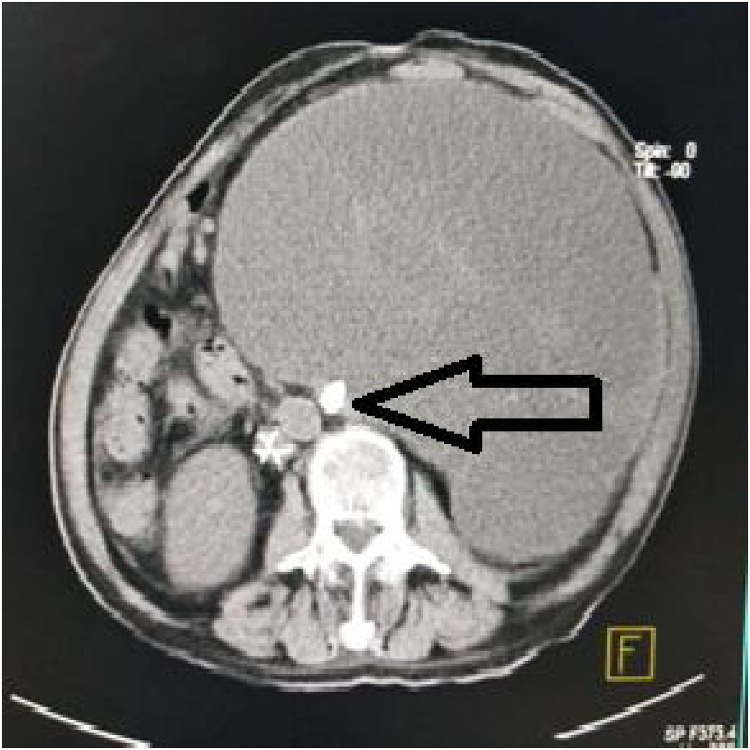
CT scan of the abdomen (transverse section) showing GH, and a high-density shadow (black arrow) located in the renal hilum region.

An 82-year-old man complained of a gradual increase in his abdominal girth over the past two years and of abdominal distension and intermittent nausea for the past six months. He was admitted to the hospital for repeated intractable hiccups having lasted two months. The patient had a history of intestinal necrosis due to an intestinal obstruction 25 years earlier and of occlusion of the inferior vena cava filter and left common iliac vein stent placement due to left common venous thrombosis five years earlier. Physical examination showed the patient was of average build with a thin, anemic appearance, and his vital signs were stable. Cardiopulmonary examination produced normal results. An abdominal examination revealed a longitudinal surgical scar in the middle of the upper abdomen. The swelling of the mass was evident on the left side of the abdomen (Fig. 1). Palpation showed the abdomen to be soft but not tender, and bowel sounds were normal. Ultrasonography and computerized tomography (CT) showed the presence of a massive hypoechoic lesion occupying almost the entire abdomen. This lesion exerted pressure on the stomach, liver, pancreas, and spleen. There was a high-density shadow visible about 1.5 cm from the middle pole of the medial edge of the mass (Fig. 2, Fig. 3). An isotope kidney scan showed poor kidney function and only 5% normal renal function in the left kidney.

**Fig. 4:**
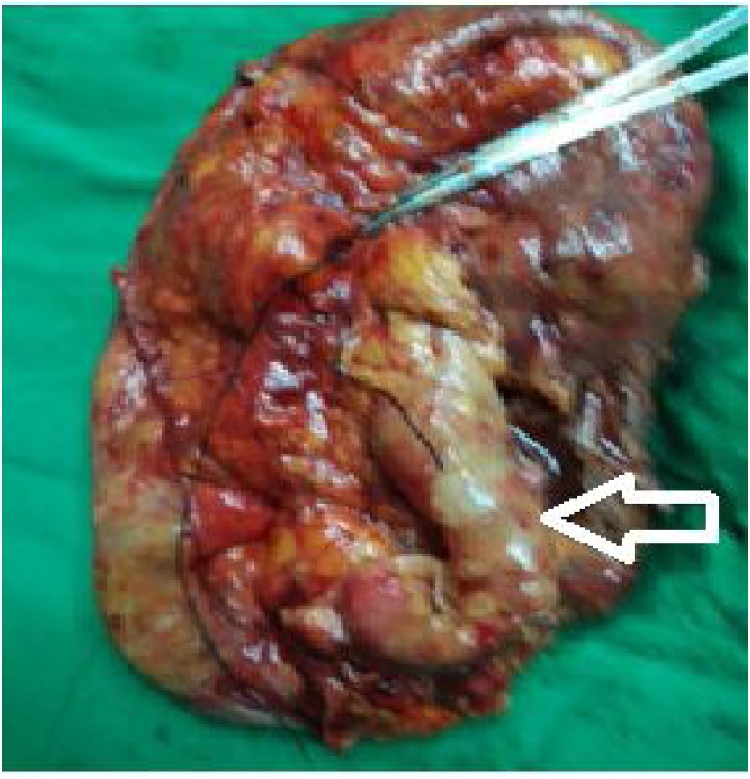
Postoperative gross pathological specimen image showing GH and dilated ureter (white arrow).

**Fig. 5:**
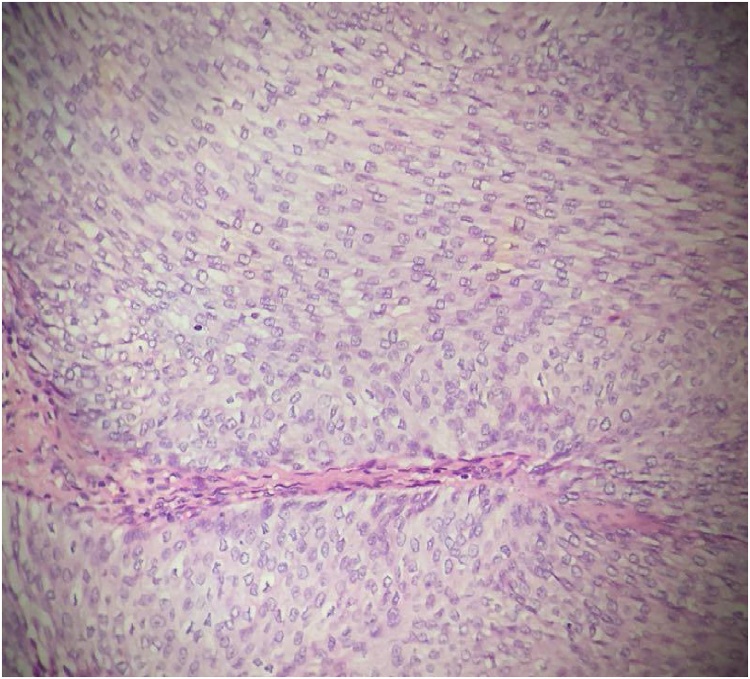
Pathological section from the GH showing an invasive papillary epithelial carcinoma (H&E staining, magnification, x200). H&E, hematoxylin and eosin.

After administration of two units of homologous red blood cells, the patient underwent what was initially laparoscopy and then open simple nephrectomy. Adhesion between the kidney and surrounding organs was evident during the procedure. About 7.5 liters of hydronephrosis fluid were drained. The left kidney and upper ureter were successfully removed (Fig. 4). The patient was diagnosed with GH caused by obstruction from a kidney stone at the junction of the ureter and renal pelvis, accompanied by renal cell transitional cell carcinoma (T2N0M0) (Fig. 5). The patient's intractable hiccup symptoms disappeared, and his condition and appetite recovered after the operation. During the six-month follow-up, the patient did not experience hiccups.

## 3 Discussion

GH is a relatively rare urinary system disorder that can occur in patients of any age, but it is extraordinarily rare in adult patients [3]. It is generally defined as hydronephrosis of 1 L in an adult or 1.5% of total body weight in a child [1,3]. Quantities of fluid in the renal pelvic system exceeding 2 L have only rarely been reported [4]. We here report an 82-year-old case of massive hydronephrosis and review the clinical features of cases of large hydronephrosis involving more than 2 L of fluid in the last decade in Table 1. GH is generally thought to develop over time, so clinical symptoms are usually not noticeable. The hydronephrosis is gradually increased to a certain extent, and it contributes to surrounding organs having compressed symptoms. Causes of GH may include UPJ obstruction, renal pelvic-ureteric calculi, pelvic-ureteric tumors, trauma, obstruction of the outlet, and obstruction of the PUJ from cross-fusion kidney or ectopic kidney [[5], [6], [7], [8]].

**Table 1:** List of cases of GH reviewed in the literature over the past decade.

Case no.	Gender	Age	Cause	Pre-operative diagnosis	Symptoms	Diagnosis methods	Treatment and drainage volume	Author
1	M	17	PUJ obstruction	Clear	Flank pain, abdominal swelling	Ultrasonography contrast-enhanced CT TC-99m EC Renal scan	PCN, nephrectomy 4L	Ashish Sharma
2	M	49	PUJ obstruction	Unclear	Malaise, suprapubic pain	Ultrasonography, CT, Radionuclide scan	Laparoscopic nephrectomy 3L	Pawel Obrocki
3	M	45	PUJ obstruction, trauma	Unclear	Flank pain, fullness, gross hematuria	Ultrasonography contrast-enhanced CT TC-99m EC	Open pyeloplasty 7.5L	Ashok Kumar Sokhal
4	F	78	Renal pelvic carcinoma, ureter stone	Unclear	Gross hematuria	Ultrasonography, contrast-enhanced CT	Open Nephroureterectomy 8.6L	Tomihiro Wakamiya
5	F	18	PUJ obstruction	Unclear	Mild abdominal pains	Ultrasonography CT	PCN 7.5L	QI-FEI WANG
6	M	20	PUJ obstruction	Unclear	Abdominal pain	CT	Open nephrectomy 8L	Guanghui Hu
7	M	83	Ureteral stone	Unclear	Abdominal flank pain	Ultrasonography CT	PCN 4L	Yalcin Golcuk
8	M	47	PUJ obstruction	Unclear	Intestinal occlusion,abdominal distension	Abdominal X-ray CT	Nephrectomy 7.8L	Issam Yazough
9	F	31	PUJ obstruction	Unclear	Pelvic cystic mass	Ultrasound, MRI	Nephrectomy 6L	Lin YJ
10	M	55	Ureteral stone, tumor renal pelvis	Unclear	Gross hematuria, left shoulder pain	Enhance CT	Radical nephrectomy 7.8 L	Kimura R
11	M	40	Obstructing renal calculus	Clear	Abdominal pain, nausea, vomiting	CT	PCN 7L	Grover CA
12	M	45	Ureteral stone	Clear	Abdominal distension, nausea	CT	Nephrectomy 5L	Chia-Chao Wu
13	M	62	Renal pelvic tumor	Clear	Abdominal fullness, pain	Ultrasonography, CT, MRI	PCN 7L	Maruyama T

M = Male; F = female; PUJ = Pelvic-ureteric junction; CT = Computerized tomography; MRI = magnetic resonance imaging, PCN = percutaneous nephrostomy.

Abbreviations: M = Male; F = female; PUJ = Pelvic-ureteric junction; CT = Computerized tomography; MRI = magnetic resonance imaging; PCN = percutaneous nephrostomy; UPJ = ureteropelvic junction.

GH progresses slowly. As described in the literature, the associated massive abdominal mass or abdominal swelling may cause pain, hematuria, recurrent urinary tract infections, or other symptoms or complications described in the literature, including nausea, fatigue or indigestion, urinary tract infection, weight loss, renal insufficiency, and even kidney breakdown (Table 1). According to the literature, GH may also, though rarely, involve severe post-traumatic hematuria, intestinal compression symptoms, gastric obstruction, or respiratory distress [5,9,10]. A review of cases of GH involving over 2 L published during the past decade showed that the dominant symptom had been massive hydronephrosis of surrounding organs [5].

As far as we know, no previous reports have shown any case of hiccups caused by massive hydronephrosis. Hiccups are an abnormal respiratory movement, a diaphragmatic spasm that occurs mainly because of vagus nerve reflex or direct stimulation of the phrenic nerve and diaphragm, which causes the diaphragm and intercostal muscle to involuntarily and synchronously contract. This instantly produces strong inspiratory movement. Intractable hiccups is defined if the attack lasts more than 1 month, and there are various factors that can cause intractable hiccups, including metabolic abnormalities, psychogenic diseases, malignant tumors, central nervous system pathology, medications, pulmonary disease, and gastrointestinal conditions [11]. In this case, the patient showed no central nervous system disease that could cause intractable hiccups. The most common symptoms were gastrointestinal and caused by massive hydronephrosis. One possible cause of intractable hiccups is that huge hydronephrosis oppresses the surrounding organ tissues. The compression of the neighboring gastrointestinal tract and diaphragm muscle stimulates the vagus nerve and the phrenic nerve, causing hiccups. The patient’s intractable hiccup symptoms did not show significant relief after treatment with various drugs, and they disappeared immediately after nephrectomy.

GH is mainly diagnosed via ultrasound examination and CT scan [12,13]. In most cases, the differential diagnosis between GH and other posterior abdominal cystic structures is still difficult, especially in patients with large hydronephrosis. The basic structure of the kidney disappeared, and, because of its large size, the hydronephrosis exerted squeezing pressure all around the organ. A review of the literature on GH over the past decade showed that only 30% (4/13) of cases could be clearly diagnosed before surgery (Table 1). As shown in this case report, a coronal-view CT examination of the patient revealed a high-density shadow of approximately 1.5 × 1.0 cm in the middle pole of the medial margin of the cyst (Fig. 2, Fig. 3). The diagnosis of left pelvic-ureteric junction stones was confirmed by gross pathology after the operation. The pathological findings concerning the papillary mass around the stone indicated renal pelvic transitional cell carcinoma (Fig. 5). Therefore, for the diagnosis of GH, considering the long-term stone obstruction, the local complications of malignant tumors should be taken into consideration. Similar studies have noted these types of pathological results [14]. Contrast-enhanced abdominal and pelvic CT can show the structure of the tissues around the tumor very well. Enhanced CT scan is the gold standard for the diagnosis of GH [15]. Other useful diagnostic imaging techniques include abdominal radiography and intravenous urography. CY-19-9 provides a new method of non-radiative exposure for GH diagnosis, and a reduction in urinary CA19-9 levels during follow-up may predict superior surgical outcomes and recovery of renal function [16].

The treatment of GH involves nephrectomy if the kidneys are nonfunctional; for functional organs, the treatment includes percutaneous nephrostomy, pyeloplasty, renal cortical fold repair, and occasionally nephrectomy [17]. Patients with advanced age and anemia may not be good candidates for surgery. Because the patient's intractable hiccups gradually increased, the patient and his family eventually selected right nephrectomy, and the symptoms had not recurred as of half a year of follow-up.

In conclusion, GH is a rare disease, and its symptoms are diverse. The rarer symptoms of cystic hypertonic compression of surrounding organs, such as intractable hiccups, should be taken into account. Treatment of GH includes percutaneous nephrostomy, pyeloplasty, renal cortical fold repair for functional kidneys, and nephrotomy for non-functional kidneys. This case and literature review showed that GH combined with malignant tumors of the renal pelvis is common, so unless it can be diagnosed as benign GH, malignant lesions should be taken into account when planning treatment, so as to prevent postoperative tumor recurrence and metastasis [18].

## Sources of funding

The authors declared that this study supported by Beijing Council of Science and Technology “Special Project for Applied Research of Capital Clinical Features” (NO Z171100001017131).

All authors have issued final approvals for the version to be submitted.

## Ethical approval

This study is exempt form ethical approval.

## Consent

Written informed consent was obtained from the patient for publication of this case report and accompanying images. A copy of the written consent is available for review by the Editor-in-Chief of this journal on request.

## Author contribution

Xiaoxing Liao performed the surgical procedure, proposed the study and wrote the paper.

Jianhua Yang collected data and pictures from surgery.

Nianzeng Xing supervised the paper and controlled all the analysis of results, including language.

## Registration of research studies

Not needed.

## Guarantor

Dr.Xiaoxing Xiao.

Dr.Nianzeng Xing.

## Provenance and peer review

Not commissioned, externally peer-reviewed.

## Declaration of Competing Interest

No conflicts of interest.
